# Calmodulin and Calmodulin Binding Proteins in *Dictyostelium*: A Primer

**DOI:** 10.3390/ijms21041210

**Published:** 2020-02-11

**Authors:** Danton H. O’Day, Ryan J. Taylor, Michael A. Myre

**Affiliations:** 1 Cell and Systems Biology, University of Toronto, Toronto, ON M5S 3G5, Canada; 2 Department of Biology, University of Toronto Mississauga, Mississauga, ON L6L 1X3, Canada; 3 Department of Biological Sciences, Kennedy College of Sciences, University of Massachusetts Lowell, Lowell, MA 01854, USA; Ryan_Taylor1@student.uml.edu (R.J.T.); Michael_Myre@uml.edu (M.A.M.)

**Keywords:** calmodulin, calmodulin binding proteins, calmodulin binding motifs, calcium signaling, EF hands, heart arrythmia, neurodegeneration, *Dictyostelium discoideum*

## Abstract

*Dictyostelium discoideum* is gaining increasing attention as a model organism for the study of calcium binding and calmodulin function in basic biological events as well as human diseases. After a short overview of calcium-binding proteins, the structure of *Dictyostelium* calmodulin and the conformational changes effected by calcium ion binding to its four EF hands are compared to its human counterpart, emphasizing the highly conserved nature of this central regulatory protein. The calcium-dependent and -independent motifs involved in calmodulin binding to target proteins are discussed with examples of the diversity of calmodulin binding proteins that have been studied in this amoebozoan. The methods used to identify and characterize calmodulin binding proteins is covered followed by the ways *Dictyostelium* is currently being used as a system to study several neurodegenerative diseases and how it could serve as a model for studying calmodulinopathies such as those associated with specific types of heart arrythmia. Because of its rapid developmental cycles, its genetic tractability, and a richly endowed stock center, *Dictyostelium* is in a position to become a leader in the field of calmodulin research.

## 1. Introduction

Calcium mediates aspects of almost all cellular functions in eukaryotes. To understand cellular evolution and the success of multicellular organisms, it is essential to understand the evolution of calcium signaling [1,2,3,4]. Calcium signaling is also a central and critical regulator of a diversity of processes and events in the model amoebozoan, *Dictyostelium discoideum*. Calcium primarily works by binding to and regulating calcium binding proteins (CaBPs) including the omnipresent and primary CaBP calmodulin (CaM). Amoebozoans are situated at the base of the Unikonta, thus, providing a unique position between Bikonta (e.g., algae, ciliates, land plants, etc.), Opisthoconta (e.g., choanoflagellates, fungi and others), and Metazoa [1]. Studying CaM in *Dictyostelium* can thus provide valuable insight into the evolution of calcium and calmodulin signal transduction.

### Dictyostelium Calcium Binding Proteins

Calcium binds to calcium binding domains. Data mining reveals that all protists, including *Dictyostelium*, possess proteins that have the same types of calcium binding domains found in both plants and animals [3]. While calcium binding predominantly occurs through EF hand sequences, as discussed below, non-EF hand binding also occurs. *Dictyostelium’s* EF hand CaBPs include CaM (CalA), calcineurin subunit B types 1 and 2 (CnbA, CnbB), centriolar centrin-B (CenB), alpha-actinin A (AbpA), frimbrin (FimA), and calfimurin-1 (Caf-1), plus many others including a host of yet uncharacterized EF-hand proteins. To be precise, *Dictyostelium* CaM will hereafter be referred to as CalA while CaM will be used in a generic sense for other organisms. In addition to these, a group of penta-EF CaBPs have five, rather than four, contiguous helix-loop-helix calcium-binding motifs; however, due to amino acid variants, some of the EF hands may be unable to bind the divalent cation. Two penta-EF hand proteins have been isolated in *Dictyostelium:* apoptosis-linked gene (ALG) 2 homologs, PefA/Alg-2a and PefB/Alg-2b but neither appears to play detectable roles during growth and development [5].

In contrast, non-EF hand calcium binding occurs via C2 and less-well-defined non-canonical calcium binding sites. As in other species, C2 binding motifs bind to calcium in a small number of proteins (e.g., copines, annexins) [6]. C2-domains are larger than any other calcium-binding region consisting of 120–130 amino acids that take on a beta-sheet sandwich comprised of two four-stranded beta-sheets connected by three calcium-binding loops [2]. Carbonyl, aspartate-side chains and water mediate the binding. A smaller group of proteins have been shown to possess non-canonical calcium binding sites in various species, but none have been yet characterized in *Dictyostelium*.

While 14 CaBPs (Cbp1-14) have been identified these are but a small part of the approximately five dozen calcium-binding proteins that have been detected in this organism (Unitprot.org; dictyBase.org). In addition to the central calcium sensor and effector CalA, there is its CaM-like relative CalB. CalB (calB; 149 aa; 16.8 kDa; DDB_G0269104) shares 50% sequence identity with CalA [7]. A non-essential protein, CalB null cells grow and develop normally generating viable spores. Myosin light and heavy chain proteins (Mlc and Mhc) commonly bind to calcium. The myosin light chains of *Dictyostelium* share similarities to and differences from CalA [8]. For example, MlcD (147aa, 16.5 kDa) is similar in size and shares 44% sequence identity but it does not bind calcium. MlcB (73aa, 8.3 kDa) is like a single lobe of CalA, while MlcB (147aa, 16.5 kDa) has two lobes but only one binds calcium.

## 2. *Dictyostelium* Versus Human Calmodulin

Other than some of the Cbp1–14 (e.g., Cbp1, Cbp4a, Cbp7) and myosin regulatory light chain proteins, CalA is the most well studied of the calcium-binding proteins. CaM is the primary sensor and effector of calcium signal transduction in eukaryotes [8]. It functions in turn by binding to over 300 different CaM-binding proteins (CaMBPs) that mediate hundreds of cellular functions [9,10,11]. Despite this, most papers on calcium function and signal transduction fail to determine if CaM is at play. Even those studies that involve well established CaMBPs, such as the various CaM kinases, commonly ignore the critical role of CaM other than to note it is part of the enzyme’s name. It is impossible to know why this lack of attention to this little regulatory molecule is so pervasive, but one reason could be its promiscuous behavior and the relative difficulty in dissecting how it binds to so many target proteins.

CalA mediates events during every stage of the asexual life cycle and many events during sexual development of *Dictyostelium discoideum* [12,13]. CaM knockouts are lethal in those species where they have been studied [14]. In 1980, Clarke et al. first reported on *Dictyostelium* CalA, comparing it to bovine CaM, a well-studied protein at the time [15,16,17]. Because *Dictyostelium* is a model system for the study of a diversity of human disease genes, many of which involve CaM, comparisons with human CaM are critical. Considering the millions of years that separate them, *Dictyostelium* discoideum CalA and Homo sapiens CaM are amazingly similar. The three-dimensional (3D) structure of CaM as well has its binding to its target proteins has been studied in numerous species (e.g., [18]). CaM has changed little through evolution such that its sequence in plants, animals and microbes differs in only a handful of amino acids. In 2001, Friedberg and Rhoads presented CaM protein sequence alignments for a diversity of eukaryotes [19].

**Figure 1:**
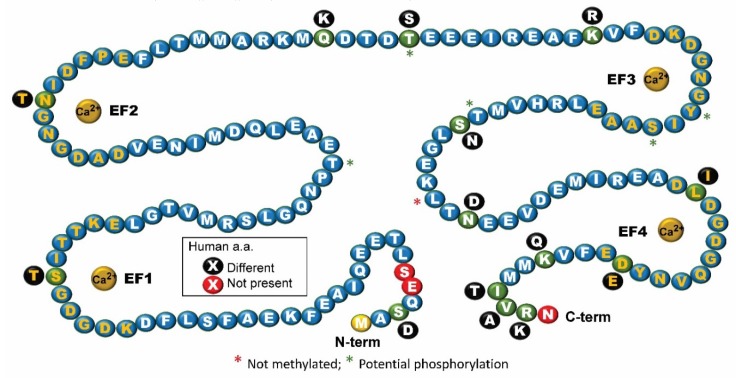
The sequence of *Dictyostelium* calmodulin (CalA) noting the differences from human calmodulin (CaM). Black circles, adjacent to the green circles in CalA, indicate amino acid differences in human CaM. Red circles show amino acids that are not present in human CaM. EF hand (EF-1 to -4) amino acids are shown in gold text. Note: the initiator methionine shown here is removed from functional CalA. Graphic modeled designed after Figure 1 in [19].

At 152 amino acids in length, *Dictyostelium* CalA (kDa 17.12) possesses three more amino acids (two in N-term, one in C-term) than human CaM (CALM1) and differs in only 13 amino acids (Figure 1). While one gene encodes *Dictyostelium* CalA (calA), humans have three different CaM genes (CALM1-3) that are spliced to generate identical CaM proteins (149aa; 16.84 kDa). Human CaM possesses multiple sites for acetylation, methylation, and phosphorylation, of which methylation and phosphorylation have been most studied [18]. While *Dictyostelium* CalA has a lysine in the same position where trimethyllysine is present in human CaM, it is not methylated. As for human CaM, the initiator methionine is removed from CalA [17]. Human CaM is phosphorylated by various kinases and all the phospho sites present in human CaM are also present in *Dictyostelium* CalA but which residues, if any, are phosphorylated in the eukaryotic microbe remain to be determined.

Human CaM possesses four calcium-binding EF loops—two in the N-term lobe and two in the C-term lobe—each spanning 12 amino acids. Single amino acid differences in three of the four calcium-binding EF hands involve amino acids from the same type. Research supports that this form evolved via two successive gene duplications of a single EF-hand sequence [20,21]. The *Dictyostelium* EF loops in CalA are: EF1, DKDGDGSITTKE; EF2, DADGNGNIDFPE; EF3, DKDGNGYISAAE; EF4, DLDGDGQVNYDE (Figure 1). The same residues at positions 1, 3, 5, and 12 (underlined) are identical with human and most other eukaryotic CaMs [21]. While calcium-binding studies have not been done with CalA, this strong homology suggests all four EF hands are each capable of binding calcium ions like human CaM. The side chains of these critical residues provide oxygen atoms involved in Ca^2+^ binding. In spite of this conservation, the EF hand pairs (E1/E2; E3/E4) have different Ca^2+^ binding affinities and kinetics with the binding status (i.e., calcium occupancy per lobe) affecting the conformation of the protein and, as a result, its ability to bind to target CaMBPs. Ca^2+^ binding is also affected by binding of CaMBPs adding further complexity to understanding CaM structure and function.

## 3. Calcium-Binding Causes Dramatic Conformational Changes in Calmodulin

**Figure 2:**
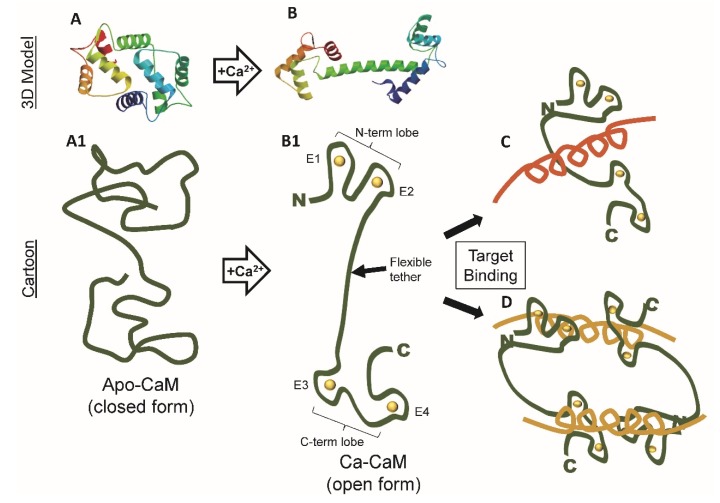
Simple line drawings depicting the fundamental CaM conformation in apo- (**A**,**A1**) versus calcium-bound (**B**,**B1**) calmodulin plus two calcium-dependent CaMBP interactions (**C**,**D**). Three-dimensional (3D) Model, *Dictyostelium* CalA (Swissprot modeling); Cartoon: Target binding examples: C, myosin light chain kinase, D: small conductance Ca^2+^-activated potassium channel (SK2).

In the presence of calcium, CaM (Ca^2+^/CaM) is in the “open position” with the calcium binding lobes distinctly separated by an extended alpha helical tether or linker region (Figure 2). In this conformation, hydrophobic residues for target protein binding are exposed in each lobe. In the absence of calcium, apo-CaM takes on a closed form at least partially occluding the hydrophobic residues, but instead allowing target binding via calcium-independent IQ, IQ-like motifs, or via less understood non-IQ motifs (Figure 2).

Essentially Ca2+/CaM can be envisioned as a two headed barbell with two calcium-binding EF hands at each end separated by a flexible alpha helical linker. While the two lobes of CaM are structurally similar, they each show distinct properties not only in binding calcium ions but also in binding target proteins [22]. The N-lobe possesses faster kinetics but lower binding kinetics while the C-lobe has slower kinetics but higher binding affinities. Calcium-independent CaMBP binding (e.g., neurogranin) primarily occurs in the closed state via the C-lobe while calcium-dependent interactions (e.g., calcineurin, CaMKII) involve both lobes in CaM’s open state. As of yet, such interactions have not been addressed in *Dictyostelium*.

Within the linker region is a hinge sequence that allows the protein to transform from the dumbbell shape to a bent configuration where the EF hands now come into closer proximity. This conformational change is induced by the presence of calcium ions. Because of the dynamic and extensive conformational events induced by first calcium binding and then CaMBP binding, CaM has been and still is actively studied by chemists, biochemists, structural, and theoretical biologists to name a few. New insights into these events continue to be revealed using new technologies (e.g., [10,23,24] Here, we will examine the fundamentals of these binding events with a view to their impact on the structure and function of *Dictyostelium* CalA.

## 4. Calcium-Dependent Calmodulin Binding

How is it possible for a single small protein to be able to specifically bind to and regulate over 300 different proteins? No other protein has been shown to bind to and regulate so many diverse targets. Despite the wealth of information that has been gleaned from CaM-binding interactions with a large number of target proteins, a unified model for CaM-CaMBP binding is still elusive [25]. While the precise steps in both apo-CaM and Ca^2+^/CaM binding are still under intense analysis, some insight has been gained. Unlike other protein-protein interactions, CaM-binding, in all studied species, does not involve a specific sequence of amino acids but, instead, various configurations of amino acid types. The binding is also dependent on the conformation that CaM has based upon the number and location of calcium ions that are bound to the N-term and C-term lobes, a subject still under scrutiny.

As indicated, there are two types of CaM binding: calcium-independent (apo-CaM) and calcium dependent (Ca2+/CaM). IQ, IQ-like, and some other unique sequences in target proteins mediate the binding of apo-CaM. In this binding mode, the absence of bound calcium makes the hydrophobic residues unavailable for binding leaving polar and charged residues open to mediate shallow binding to calcium independent CaMBPs [24]. Deeper binding occurs with calcium-dependent CaMBPs where calcium binding opens CaM making certain hydrophobic amino acids available for protein-protein interactions. Westerlund and Delemotte’s data suggest that the N-lobe initiates fast and flexible binding while C-lobe binding leads to deeper, more selective, hydrophobic-mediated target binding [24]. Other research has revealed that differences exist in N-lobe and C-lobe binding and these binding events are calcium- and CaMBP-dependent as indicated above.

### Calmodulin Binding Domains and Motifs

When bound to calcium, key flexible methionine residues in CaM become available for target protein binding. This, coupled with the flexibility of the central linker sequence, allows Ca^2+^/CaM to interact in a diversity of ways, via a multitude of binding domains with hundreds of target CaMBPs. The question remains: how can a loose association of various hydrophobic amino acids lead to specific binding to calmodulin? Considering that cell survival depends on successful CaM function, this is not a minor question. Moreover, a full understanding of this binding will unlock many biomedically important areas for pharmaceutical development.

While many CaM binding regions have been defined in many proteins in a diversity of species, the functional CaMBDs within those regions have received much less attention and only a comparative few have been critically analyzed. In *Dictyostelium*, calcium-dependent CaMBDs bind via sequences that fall into two general categories: canonical and non-canonical binding motifs (Table 1).

The canonical binding motifs are primarily characterized by the relative positions of hydrophobic residues. Potential motifs can be recognized by programs such as the Calmodulin Target Database after which deletion constructs and labelled peptide equivalents can be used to validate them as detailed below. The Calmodulin Target Database has a greater than 90% prediction rate for identifying valid CaMBDs [14,31]. New methods for detecting CaMBDs have recently been developed but have yet to be utilized widely and their precision remains to be verified. For both canonical and non-canonical domains, deletion constructs are typically used to validate identified binding domains. Both approaches have been used by various research teams in *Dictyostelium* to define the specific motif or simply its general localization within the putative binding protein (Table 1).

In 2008, Catalano and O’Day listed all the experimentally validated and putative CaMBDs in *Dictyostelium* proteins up to that year [14]. The types of binding motifs within them were also revealed [14]. Since then, numerous new CaMBPs possessing putative CaMBDs have been found and, within them, various binding motifs have been defined [13]. In Table 2, proteins that possess specific CaM binding motifs are listed next to each motif type. These early data suggest that only a few motifs predominate (i.e., are found in a diversity of proteins): 1-10, 1-5-10, 1-14, and 1-16. It should also be noted that for several proteins multiple motifs are present in individual CaMBDs, which likely increases their likelihood of being functional CaMBDs [13]. Despite this, no studies have been carried out in *Dictyostelium* to determine which residues and which motifs are involved in the actual binding of a validated CaMBP to CalA.

The *Dictyostelium* CaMBPs studied to date contain validated or potential canonical CaMBDs. To our knowledge, no algorithm exists to find putative non-canonical CaMBDs.

## 5. Calcium-Independent Calmodulin Binding via IQ Motifs

The closed apo-CaM configuration binds to IQ ([FILV]Qxxx[RK]Gxxx[RK]xx[FILVWY]) or IQ-like ([FILV]Qxxx[RK]Gxxxxxxxx) motifs (Table 3). IQ and IQ-like motifs mediate binding to CaM and various CaM-related light chains (e.g., myosin light chains). Less than a dozen non-IQ motifs have been found in *Dictyostelium*. Ca^2+^-independent binding motifs are alpha-helical regions devoid of prolines. While the primary focus has been on revealing calcium-dependent CaMBPs, IQ mediated calcium-independent CaM-binding has been actively studied for the myosin-I family where all three types of calcium-independent binding have been found (Table 3).

Consisting of an N-terminal ATPase motor domain (i.e., head), a light chain-binding neck, and a tail, class I myosins do not form bundles. Instead, as individuals, they move actin filaments along cell membranes in eukaryotes. In *Dictyostelium* they are involved in chemotaxis, cortical tension, macropinocytosis, phagocytosis and vesicular movements. Myosin light chains are CaM and CaM-related family members that bind to IQ motifs present in the neck domain of myosins [32,33]. Typically, a series of IQ motifs are arranged in tandem separated by 20–27 amino acids. Starting with the domain nearest to the myosin motor domain, they are numbered in sequence from IQ1, IQ2, etc. The structural integrity of the myosin holoenzyme is dependent on the IQ-mediated binding of the light chains which can also serve some regulatory functions.

Seven myosin-I isozymes are expressed in *Dictyostelium* [34]. Three have short tails (MyoA, MyoE, and MyoF), three have long tails (MyoB, MyoC, MyoD), while MyoK lacks both a neck and tail (i.e., no light chains present). Except for MyoF, the light chain binding attributes of all of the myosin-I isozymes of *Dictyostelium* have been revealed with only two using CaM as a light chain. The two IQ motifs present in both MyoA and MyoE both bind CaM, while MyoC does not bind CaM but instead binds the light chain MlcC [34]. The non-traditional binding between MyoC and MlcC is mainly due to specific hydrophobic residues (Phe-718, Tyr-736) that have replaced arginine found in traditional IQ motifs [35]. Although myosin-I (e.g., myosin-1A) isozymes have been shown to use CaM as a light chain in vertebrates, MyoA and MyoE are the first *Dictyostelium* forms of myosin-I to be shown to use CaM in this manner. Furthermore, each of the IQ motifs of MyoA and E both possess 1-5-8-14 hydrophobic acid residues, a canonical Ca^2+^-dependent CaM-binding motif as discussed above. The significance of this binding remains to be elucidated.

To date, no other Ca^2+^-independent CaMBPs have been shown to possess full IQ binding motifs but only to possess IQ-like motifs (Table 3). For example, four IQGAP family members have been identified in *Dictyostelium* each possessing a functionally unverified IQ-like domain (IQ/LQ): DGAP1/DdIQGAP1, GAPA/DdIQGAP2, and DdIQGAP3 [36,37]. At least two proteins contain both Ca^2+^-dependent CaMBDs and IQ motifs (e.g., NumA, VatM), and thus, can bind to CalA in the presence and absence of calcium.

## 6. Detecting Calmodulin-Binding Proteins

Over the years a number of methods have been employed to identify populations of CaMBPs as well as to isolate specific proteins. Early ^125^I-Calmodulin experiments demonstrated associations with calcineurin (*CanA*) and the large(60S) ribosomal subunit L19 (*Rpl19*) [26,38]. Population analyses of CaMBPs in *Dictyostelium* were carried out during both sexual and asexual development using the CaM Binding Overlay Technique (CaMBOT) that utilizes ^35^S-radiolabelled CaM [39,40,41]. Several dozen individual calcium-dependent and over one dozen calcium-independent CaMBPs were detected, many of which could be linked to specific events such as folic acid- or cAMP-mediated chemotaxis and to biomembrane fusion among others. Subsequent probing of DNA expression libraries of *Dictyostelium* using CaMBOT led to the identification of many proteins not previously known to be CaMBPs including histone H1 (*H1*), phosphoglycerate kinase (*PgkA*) and thymidine kinase (*TK1*) and others [14]. Two other CaMBPs identified by CaMBOT—the nucleolar number regulator nucleomorphin (*NumA*) and the secreted, cysteine-rich matricellular protein (*CyrA*) have led to many other central discoveries including new binding partners, protein translocations and functions including the role of extracellular calmodulin [27,28,29,30,42,43,44]. A later CaMBP probing method utilized a glutathione-S-transferase (GST-CaM) fusion assay revealed DwwA, an IQ-like-motif protein required for scission in cytokinesis, similar to the human KIBRA [45]. More traditional approaches, CaM co-immunoprecipitation pulldown assays using anti-CaM antibodies or CaM conjugated agarose identified cyclin-dependent kinase 5 (Cdk5), a cytoplasmic and nuclear serine-threonine kinase with multiple transport and cell cycle roles; and the cell-cell adhesion molecule CadA [27,46,47].

A diversity of studies have used CaM antibodies to colocalize various proteins with CalA, coimmunoprecipitate them, and/or isolate CaMBPs in *Dictyostelium*. In keeping with this, immunofluorescent experiments showed strong co-localization of CalA with the nucleoside diphosphate kinase (NDPK) on intracellular vesicles suggesting a role for CalA in mediating pinocytosis [48]. The unique, developmentally necessary huntingtin protein (HTT) of Huntington’s disease, has emerged as a possible CaMBP [49]. Cytoskeletal and chemotaxis-related proteins feature prominently, from myosin-II-driving Myosin heavy chain kinases (MhkA, MhkB) and P21-activated kinases (MiHCK/PakB, PakA), to the Ras GTPase-activating-like protein (rgaA) [13,14]. As also detailed in those reviews and Table 1, major signal transduction receptors reported include the chemotactic folic acid receptor (fAR1) and Ca^2+^-signaling inositol 1,4,5-trisphosphate receptor-like protein A (IplA). Suspected CaMBPs extend to the network of the osmoregulatory contractile vacuole (CV), with Ras-like RABs (Rab8a, Rab11a, Rab11c), Rh-like ammonia transporters (RhgA, RhgB), the Vacuolar ATPase subunit M (VatM), Von Willebrand factor kinase (VwkA), and the phagocytic drainin (phgA). Lastly, proteins of the cellular degradative processes of autophagy (Atg1, Atg13, Atg101) also appear to have CaM binding motifs. The range of actual and potential CaMBPs emphasizes the functional and regulatory importance of calmodulin, as well as the need for confirmation of CalA-CaMBP relationships.

Once a candidate is found, its CaM-binding needs to be validated using at least two different interaction methods (e.g., immunoprecipitation, CaM-agarose). CaM-binding is further explored in more detail, creating and assessing CaMBPs in which the predicted binding site has been mutated or deleted. Moreover, when an interaction site has been identified, specific blocking peptides can be synthesized and used in excess during pulldown experiments to perturb the interaction of CaM and the CaMBP of interest. This is often performed in parallel with a control scrambled peptide to verify the specificity and interaction kinetics of the CaMBP binding domain. When these are validated, the protein can be called a true CaMBP and its protein associates can them be searched for to determine how the CaMBP is involved in the CaM-dependent process under study.

## 7. Calmodulin Antagonists Provide Some Insight

Since it appears to be impossible to produce null-CaM strains, initial investigations into the potential role of calmodulin in a cellular process often employ calmodulin antagonists. A number of validated antagonists are effective in *Dictyostelium* (e.g., W5, W7, calmidazolium, melittin, trifluoperazine, and others). Calmodulin antagonists, erroneously called inhibitors, work by interfering with binding to CaMBPs such as brain cyclic nucleotide phosphodiesterase and have been used to treat *Dictyostelium* cells since 1981 [16]. The most used antagonists have been calmidazolium, trifluoperazine, W-5 (N-(6-aminohexyl)-1-naphthalenesulfonamide hydrochloride), and W-7 (N-(6-aminohexyl)-5-chloro-1-naphthalenesulfonamide). Their use has initiated our understanding of the function of calmodulin in the uptake of vesicular calcium ions, cAMP oscillations, chemotaxis, stalk cell differentiation, spore germination, and a host of other events [13,40,50,51,52,53]. CaM antagonists do not inhibit CaMBPs. Only a few inhibitors of specific CaMBPs have been identified and studied in *Dictyostelium*. The immunosuppressant cyclosporin and FK508 were used in early studies revealing the importance of calcineurin in asexual development, work that has continued on this central CaMBP [54,55].

## 8. *Dictyostelium* as a Potential Model to Study Calmodulin Mutations

While CaM knockouts have never been detected as the basis of any medical condition, several recent studies have reported mutations in the CALM genes of human patients [19,56,57,58]. These include mutations that alter calcium ion binding affinity to the protein that are associated with important signaling events in heart rhythm during infancy as well as epilepsy and delayed neurodevelopment. The finding that CaM mutations exist is of extreme importance with respect to how we view CaM function. All known mutations (~17) are linked to cardiac phenotypes, suggesting that the cells of the heart are particularly sensitive to CaM mutations, whereas other cell types and signaling pathways regulated by CaM appear to be less affected. However, there is much more research to be done in this area to determine how many more mutations might exist and if so, are they specifically detrimental to other critical organs. The clinical phenotypes that are a result of different CaM mutations include long-QT syndrome (LQT), catecholaminergic polymorphic ventricular tachycardia (CPVT), and idiopathic ventricular fibrillation (IVF) have been reviewed [19,58,59].

**Figure 3:**
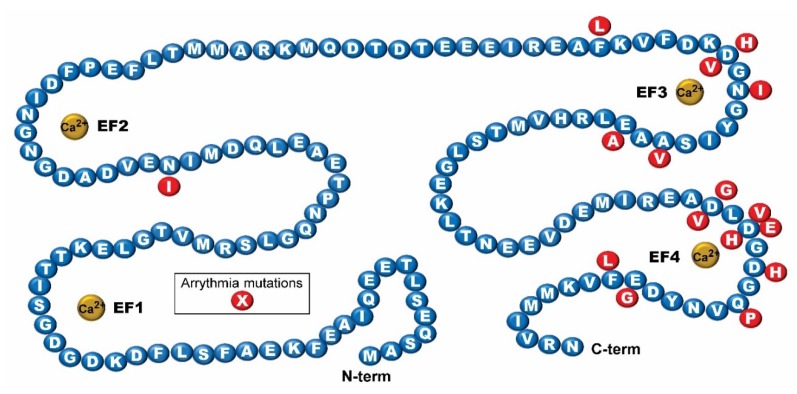
The sequence of *Dictyostelium* calmodulin showing the location of human calmodulin mutations associated with various arrythmias. Red circles indicate amino acids involved in these calmodulinopathies as detailed in the text. Graphic designed after Figure 1 in [19].

Mutational hot spots appear to occur at specific residues predominantly in EF hands 3 and 4 found in the C-terminus of the three human genes (CALM1-3). The location of these mutations in *Dictyostelium* CalA is shown in Figure 3. *Dictyostelium* is a genetically tractable haploid model organism that is amenable to transformation with dual expression vectors, gene knock-down or knock-in and most recently Clustered Regularly Interspaced Short Palindromic Repeats (CRISPR) technology [60]. Furthermore, a new dual single guide RNA (sgRNA) expression vector for use in *Dictyostelium* allows one to simultaneous insert two sgRNAs via a one-step cloning procedure [60]. By applying this system, precise point mutations specific for *Dictyostelium* CalA that model human mutations can easily be made in clonal cell lines and analyzed for effects on growth, cytoskeletal arrangement, chemotaxis, and many other conserved cellular processes. *Dictyostelium* thus presents a novel model system to potentially analyze both the impact of CaM mutations on the physiology of the cell, as well as structure-function assays that may shed light on the impact these mutations have on the conformation of calmodulin both in the absence or presence of calcium.

## 9. CaM Kinases and Calcineurin

CaM regulates a multitude of biological processes from cell movement and proliferation to metabolism and learning and memory. One critical role of CaM in cellular function can be illustrated by the opposing functions of two well-studied CaMBPs: Calcineurin and CaMKII. Calcineurin is the sole calmodulin-dependent protein phosphatase in eukaryotes, while CaMKII is a CaM-dependent protein kinase. Their Yin-Yang functions dominate a diversity of calcium-mediated signaling pathways, including the regulation of dendritic spine morphology, cell proliferation, and apoptosis to learning and memory as has been reviewed [61]. While *Dictyostelium* possesses calcineurin (*CanA*), it lacks a canonical CaMKII [26,62]. In fact, while animal cells possess a large diversity of CaM-regulated kinases, *Dictyostelium* has only one (VwkA) that has been verified and there is no evidence that it works in concert with calcineurin [63]. Five other *Dictyostelium* kinases have conserved CaMBDs, thus suggesting potential CalA-binding, but none have been proven to be regulated by CaM. An IQ-containing LISK family kinase is also present but uncharacterized. When in evolution did the CaM kinase population increase and when did the CaMKII\calcineurin interaction develop? CaM-dependent protein phosphorylation is intimately linked to the CaM-dependent events of both folic acid and cAMP chemotaxis suggesting that more work needs to be done on CaM-dependent kinases [40]. This is critical for ongoing attempts to catalog the *Dictyostelium* kinome [62].

## 10. Conclusions, Comments, and Speculation

There is little doubt that calcium was the first regulator of cell function [2,3,4]. Was the subsequent evolution of CaM in eukaryotes the quintessential event that set the stage for the multicellular development of plants and animals? As early as 1987, Swan et al. showed protein S from *Myxococcus xanthus* contains four EF hand motifs similar in sequence to eukaryotic calmodulin, suggesting that “the EF-hand super-family may have evolved from ancient proteins” found in prokaryotes [64]. The unique positioning of the amoebozoan *Dictyostelium* at the base of the unikonta and its central role as a model research organism argues that it can yield valuable information on the evolution of both calcium signaling and the central role of calmodulin in the animal kingdom. Because of its wide appeal as a biomedical model and the highly conserved structure of its CaM, *Dictyostelium* is also in a strong position to provide unique insight into the regulatory functions of this essential protein. The ease of generating mutants and analyzing protein function in a haploid cell presents unique advantages over many other organisms. The extremely low comparative costs for generating and maintaining mutants and rapid timeline for data generation and analysis are also beneficial.

*Dictyostelium* will continue to provide unique insight into CaM-dependent biological processes such as autophagy, cell cycle, chemotaxis and motility, osmoregulation, cell differentiation, and others [13]. Studies on CaMBPs involved in neurodegenerative disease, including Huntington, Batten’s, and Alzheimer’s disease, are already yielding valuable insights in this model microbe [65]. *Dictyostelium* has also proven its value in the study of IQ-mediated binding of a diversity of myosin light chain molecules [34,35,66]. Future work might focus on defining the functional residues involved in calcium-mediated CaM-binding as well as their role in mediating CaM-dependent events. In addition, based on results from biophysical and chemical analyses in other systems, mutant CaMs could be generated to validate their significance in biological processes and calmodulinopathies, thus, serving as a critical interface between theoretical analyses of CaM structure and binding modes with their functional significance.

## Figures and Tables

**Table 1:** Calcium-dependent CaM-binding domains of *Dictyostelium* CaMBPs.

I. CaMBPs with Canonical CaM-Binding Domains (CaMBDs).				
**CaMBP**	**ORF/Dictybase**	**CaMBDs**	**Verified**	**Reference**
Atg13	DDB_G0269162	104FYKNVIILIRTIYAML129	N	[13]
Atg101	DDB_G0288287	72LTSIMKRKAKTAQISI86	N	[13]
CanA/CAN	DDB0185021	445RQMLRAKVKSVSKMMRMFSLLRQE468	Y	[26]
		529LLRMNSRGELFRINSKGDLF548	N	[14]
Cdk5	DDB_G0288677	17IVYKAKNRETGEIVALK33	Y	[27]
		132LLINRKGELKLADFGLARAFGIP154	N	[27]
cmbB	DDB0201645	179IPKSLRSLFLGKGYNQPLEF198	N	[13]
DDB_G0285767	DDB_G0285767	172FFEYRENLIKTANKSF191	N	[13]
		240WYKHLKEEFSKVKLKV255	N	[13]
Drainin	DDB_G0269130	380RTALSILRYFIS391	N	[13]
fAR1	DDB_G0281211	611CVLIIFGAKFWKIYKPVEDD630	N	[13]
Htt	DDB_G0272344	1885LDLRKKQLLRLLSL1896	N	[13]
IplA	DDB_G0292564	841VSKGRNYNGI850	N	[13]
		850IRLVGQRITHKECL863	N	[13]
MIHCK/pakB	DDB0191345	150AFRKAYHTLDLSKSGASGRY169	N	[14]
MHCK-A/mhkA	DDB0216274	535FVSLARIVKINVGTREIRV553	N	[14]
MHCK-B/mhkB	DDB0191333	150AFRKAYHTLDLSKSGASGRY169	N	[14]
*NdkC*	DDB_G0273069	23GLVGEIIARYEKKGFVLVGLKQLV46	N	[13]
NumA	DDB0231257	171EDVSRFIKGKLLQKQQKIYKDLERF195	Y	[28]
PakA/ DPAKa	DDB0191313	291EIEKIQREERIKIEKEYDDK310	N	[14]
PatA	DDB_G0277861	1064WQIVRQTHKKLVVINALKE1085	N	[13]
PgkA	DDB0191349	209KPFLAILGGAKVSDKIKLIF228	Y	[29]
PsaA	DDB_G0270994	108LSLVFTGLLNDKLKGFYRSKYTV130	N	[30]
Rab11A	DDB_G0269238	69TAGQERYRAITSAYYRGAVGALLV92	N	[13]
Rab11C	DDB_G0277101	68GQERFRAVTSGYYRGAVGAMI88	N	[13]
RhgA	DDB_G0283389	425VILLQLKKIKGLKSKEY442	N	[13]
RhgB	DDB_G0280059	431ILKALKKVGGLKAKQYY447	N	[13]
rgaA	DDB0191437	267MIITYNKRKQGTDYLKAVIG286	N	[14]
Rpl19	DDB0214854	123LYLKAKGNVFKNKRTLIEYIV143	N	[14]
ThyB/DdTK1	DDB0191436	20GKTTELIRRIKRFNFANKKC30	N	[13]
VwkA	DDB0216405	420LGIDEHGKKVVLKQSKYIGGR440	N	[14]
		536SKNKAIVVDIQGVKTSKGYLL556	N	[14]
**II. CaMBPs with Non-Canonical CaMBDs**				
Atg1	DDB_G0292390	110EKALYFMKQLAN121	N	[13]
Rab8A	DDB_G0280043	71TAGQERFRTITTAYYRGAMGI91	N	[13]
VatM	DDB_G0291858	291DHKRQTLAGIV301	N	[13]

Atg1, Atg13, Atg101, autophagy protein 1, 13, 101; Cdk5, cyclin-dependent kinase 5; CanA, calcineurin A; fAR1, folic acid receptor 1; Htt, Huntingtin disease protein homolog; IplA, inositol 1,4,5-trisphosphate receptor-like protein A; NumA, nucleomorphin; PgkA, phosphoglycerate kinase A; ThyB, thymidine kinase B; VwkA, von Willebrand factor kinase A; MIHCK, myosin I heavy chain kinase; MHCK-A, myosin heavy chain kinase A; MHCK-B, myosin heavy chain kinase B; *NdkC, nucleotide diphosphate kinase*; PaKa, p21-activated protein kinase A; *PatA, P-type ATPase; PsaA*, puromycin-sensitive aminopeptidase PsaA; *RabIIA, IIC, 8A*, ras-like in rat Brain GTPase IIA, 11C, 8A; RgaA, ras GTPase-activating-like protein A; RhgA, B, Rhesus-like glycoprotein; Rpl19, ribosomal subunit protein L19;VatM, V-ATPase subunit M.

**Table 2:** Calcium-dependent Calmodulin Binding Motifs.

Subclass	Motif	Proteins with Motif
***1-10 Subclasses***		
1-10	(FILVW)xxxxxxxx(FILVW)	Atg13; CanA; Cdk5; CmbB; fAR1; IplA; *NdkC*; *PatA*; PsaA; *Rab11A*; *Rab11C*; *RhgA*; *RhgB*; VwkA
1-5-10	(FILVW)xxx(FAILVW)xxxx(FILVW)	fAR1; *NdkC*; NumA; PgkA; *Rab11a*; *Rab11a*; *RhgB*; ThyB
Basic 1-5-10	(RK)(RK)(RK)(FAILVW)xxx(FILV)xxxx(FILVW)	L9
***1-12 Subclass***		
1-12	(FILVW)xxxxxxxxxxXXxx(FILVW)	Cdk5; PsaA; RhgB
***1-14 Subclasses***		
1-14	(FILVW)xxxxxxxxxxxx(FILVW)	Atg101; CanA; Cdk5; fAR1; IplA; *NdkC*; NumA; *PatA*; PsaA; *Rab11a*; *Rab11a*; VwkA
1-8-14	(FILVW)xxxxxx(FAILVW)xxxxx(FILVW)	Htt; L9; PgkA; *Rab11a*
Basic1-8-14	(RK)(RK)(RK)(FILVW)xxxxxx(FAILVW)xxxxx(FILVW)	none?
1-5-8-14	(FILVW)xxx(FAILVW)xx(FAILVW)xxxxx(FILVW)	none?
***1-16 Subclasses***		
1-16	(FILVW)xxxxxxxxxxxxx(FILVW)	Atg1; Atg13; Atg101; Cdk5; CmbB; fAR1; L9; *NdkC*; NumA; PsaA; *Rab11a*; RgaA;VwkA

***Non-canonical*:** Atg1 (110EKALYFMKQLAN121); Atg9 (221IANRIMRK228); Drainin (380RTALSILRYFIS391); Rab8a (71TAGQERFRTITTAYYRGAMGI91); VatM (291DHKRQTLAGIV301). Proteins were first scanned at the at the Calmodulin Target Database (*http://calcium.uhnres.utoronto.ca/ctdb/ctdb/home.html*) to detect putative CaM-binding domains (CaMBDs). Identified CaMBDs where then manually assessed for the presence of canonical binding motif subclasses and for non-canonical binding.

**Table 3:** Calcium-independent IQ, IQ-like, and non-IQ.

Calmodulin-Binding Motifs in *Dictyostelium*			
**Full IQ motifs ((F/I/L/V)Qxxx(R/K)Gxxx(R/K)xx(F/I/L/V/W/Y))**			
CaMBP (gene)	Dictybase	IQ	Binding Sequence
MhcA (*mhcA*)	DDB0191444	IQ1	IQAA TRGWIARKVYK
Myo1E(*myoE*)	DDB0216200	IQ1	IQKTWRGYRARSKWN
Myo1F(*myoF*)	DDB0220021	IQ1	IQRVWRGYKVRKWYD
Myo5A(*myoH*)	DDB0233685	IQ1	IQKIWRGYTDRKAYI
Myo5B(*myoJ*)	DDB0185050	IQ1	IQKRWKGYLYRKRYK
**IQ-like motifs ((F/I/L/V)Qxxx(R/K)xxxxxxxx)**			
DwwA (*dwwA*)	DDB0216188	IQ1	IQRT FRNHKKQSY
MhcA (*mhcA*)	DDB0191444	IQ2	IQQNLRAYIDFKSW
Myo1A(*myoA*)	DDB0215392	IQ2	IQRT YRGWLLVRECV
Myo1B(*myoB*)	DDB0191351	IQ1	IQKAFRNWKAKKHSL
Myo1C(*myoC*)	DDB0215355	IQ3	IQGYFRAWKQASPFF
Myo1E(*myoE*)	DDB0216200	IQ2	IQLF YRSYRYKKWFR
Myo5A(*myoH*)	DDB0233685	IQ2	FQSL IRSYLQQLEYN
Myo5A(*myoH*)	DDB0233685	IQ3	LQSL IRTNELEKQFN
Myo5A(*myoH*)	DDB0233685	IQ4	FQSL LRRLEDSKEFN
Myo5A(*myoH*)	DDB0233685	IQ5	IQSLWRSNLAKKQLK
Myo5B(*myoJ*)	DDB0185050	IQ2	IQTK LRSVHAKQQLS
Myo5B(*myoJ*)	DDB0185050	IQ3	IQKVWRAHRDRVQYQ
Myo5B(*myoJ*)	DDB0185050	IQ4	IQTVMRRHLFSEQVH
Myo5B(*myoJ*)	DDB0185050	IQ5	LQTK IRQILSKREVD
Myo5B(*myoJ*)	DDB0185050	IQ6	IQARWRMKLAKRVYI
MyoG (*myoG*)	DDB0232322	IQ1	IQSTWRMYLIRKRFI
MyoG (*myoG*)	DDB0232322	IQ2	IQKNTRRWLVQKRYQ
MyoG (*myoG*)	DDB0232322	IQ4	IQTHLRSLLSKDYSY
MyoI (*myoI*)	DDB0185049	IQ1	IQSVWRMYRCKKRYQ
MyoI (*myoI*)	DDB0185049	IQ4	VQNNIRSFIARRHSR
MyoM (*myoM*)	DDB0191100	IQ1	IQAF FKMIKIRNQYK
MyoM (*myoM*)	DDB0191100	IQ2	LQTL IRAQRAKKDFE
NumA (*numA*)	DDB0231257	IQ1	LQKQQKIYKDLERF
RgaA (*rgaA*)	DDB0191437	IQ1	IQEL KRNLVSEVRR
RgaA (*rgaA*)	DDB0191437	IQ2	LQTE PKYLAGLVYL
VatM			IQLA LRTATTRSGA
**Non-IQ motifs**			
Myo1A(*myoA*)	DDB0215392	IQ1	IGSVWKMYKQRIKWYL
Myo1C(*myoC*)	DDB0215355	IQ1	IKNAYRNYKAFQFEC
Myo1C(*myoC*)	DDB0215355	IQ2	IKNAFRNYKLYRQRC
Myo1D(*myoD*)	DDB0191347	IQ1	LQRFFLRFTLMSYYY
Myo1F(*myoF*)	DDB0220021	IQ2	IQTYYLRYKVLTYIK
MyoG (*myoG*)	DDB0232322	IQ3	LESFSRMVIFRAPYL
MyoI (*myoI*)	DDB0185049	IQ2	LGAAMLSHSSRRDFQ
MyoI (*myoI*)	DDB0185049	IQ3	IKGFFKMLTYQKQFK

The critical amino acids in the motifs are highlighted in green. Revised and updated from [13,14].
